# Brain magnetic resonance imaging and histopathology findings in a dog with global brain ischaemia following cardiopulmonary arrest

**DOI:** 10.1111/avj.13178

**Published:** 2022-06-02

**Authors:** J Goh, LM Eramanis, M Milne, A Stent, M Boller

**Affiliations:** ^1^ U‐Vet Animal Hospital, University of Melbourne 250 Princes Highway Werribee Victoria 3030 Australia; ^2^ Small Animal Specialist Hospital Level 1, 1 Richardson Place North Ryde New South Wales 2113 Australia; ^3^ VetCT 185‐187 High Street, Suite 11 Ground Floor Fremantle Western Australia 6160 Australia; ^4^ Gribbles Veterinary Pathology Clayton Victoria Australia; ^5^ Central Victoria Veterinary Hospital, VCA Canada 760 Roderick Street Victoria British Columbia V8X 2R3 Canada

**Keywords:** cardiopulmonary arrest, global brain ischaemia, histopathology, magnetic resonance imaging, post‐CPA

## Abstract

**Background:**

Global brain ischaemia following cardiopulmonary arrest is uncommonly reported in veterinary medicine yet neurologic injury after arrest is a known morbidity.

**Case report:**

An 18‐week‐old male entire Cavalier King Charles Spaniel‐Poodle was referred following 3 days of neurologic abnormalities after cardiopulmonary arrest. After resuscitation, the animal had decerebrate rigidity, a stuporous mentation and intermittent episodes of vocalisation and apnoea. A brain magnetic resonance imaging (MRI) was undertaken 4 days after cardiopulmonary arrest, with standard sequences (T1‐weighted, T2‐weighted and fluid‐attenuated inversion recovery) as well as diffusion‐weighted imaging to better discern ischaemic injury and cytotoxic oedema for prognostic reasons. MRI findings were consistent with global brain ischaemia affecting the hippocampus, cerebellum and substantia nigra, the latter two not previously identified in canine cases of global brain ischaemia. The patient was euthanased on day eight post‐cardiopulmonary arrest due to a lack of neurological improvement and developing sepsis as a complication. Ante‐mortem identification of affected areas of the brain was confirmed on histological examination, with evidence of ischaemic injury seen in the cerebrum, hippocampus, cerebellum, basal nuclei and thalamus.

**Conclusion:**

This report describes ante‐mortem MRI and postmortem findings in a dog with global brain ischaemia following cardiopulmonary arrest. A multimodal approach to neuroprognostication in patients recovering from cardiopulmonary arrest is recommended.

**Abbreviations**
ADC: apparent diffusion coefficient; ALT, alanine aminotransferase
BAER: brainstem auditory evoked response
CPA: cardiopulmonary arrest
CPR: cardiopulmonary resuscitation
CT: computed tomography
DWI: diffusion‐weighted imaging
EEG: electroencephalogram
FLAIR: fluid‐attenuated inversion recovery
GBI: global brain ischaemia
MRI: magnetic resonance imaging
ROSC: return of spontaneous circulation
SSEP: short‐latency somatosensory evoked potentials
T1W: T1‐weighted
T2W: T2‐weighted

Hypoxic‐ischaemic brain injury following cardiopulmonary arrest (CPA) is one of four components of the post‐CPA syndrome alongside myocardial dysfunction, systemic ischaemia‐reperfusion injury and the unresolved precipitating disease that lead to arrest.^1^, ^2^ Despite increasing research in veterinary cardiopulmonary resuscitation (CPR), survival to discharge rates of dogs and cats that achieve return of spontaneous circulation (ROSC) remain low at 5%–19%.^3^, ^4^, ^5^ The majority of all animals with ROSC are euthanized.^4^ Research focussing on the optimisation of care and methods of prognostication in this post‐CPA period could improve survival.^6^

The prognosis of global brain ischaemia (GBI) in dogs after CPA is difficult to determine due to a scarcity of literature on the topic. In people, prognostication focuses on certainty of predicting a poor outcome to justify withdrawal of life support. A multimodal approach is recommended that includes physical exam, electrophysiologic measurements, biomarkers and neuroimaging.^2^, ^6^ In veterinary medicine, three case reports have described magnetic resonance imaging (MRI) findings in three dogs and one cat with GBI.^7^, ^8^, ^9^ In these cases the hypoxic‐ischaemic injury was associated with anaesthesia. Two case reports included more advanced MRI sequences such as diffusion‐weighted imaging (DWI) with concurrent apparent diffusion coefficient (ADC) maps to confirm ischaemia in the cerebrum of a dog with multiple organ dysfunction^8^ and a dog post‐strangulation.^10^ Clinical case reports of post‐CPA dogs are limited to either clinical findings^11^ or brain histology findings.^12^ A case report from South Korea describes brain computed tomography (CT), MRI and histopathology findings in a post‐CPA Beagle 12 days after resuscitation, though a full English translation is unavailable.^13^

Linking ante‐ and post‐mortem findings in the canine brain helps us to understand the extent of ischaemic neurologic damage following CPA. Overall, more information on all diagnostic modalities, including physical exam, electrophysiology, biomarkers and neuroimaging, are needed on neurologic prognostication after initial resuscitation from CPA. This case report describes the neurologic examination findings coupled with brain MRI and histopathology in a dog resuscitated from naturally occurring CPA.

## Case summary

An 18‐week‐old, male entire Cavalier King Charles Spaniel‐Poodle cross weighing 2.5 kg initially presented to the referring veterinarian for acute onset of vomiting, with no history or evidence of neurological disease at that time. Due to concern for a gastrointestinal foreign body, the referring practice conducted an abdominal ultrasound under general anaesthesia as it was deemed necessary for adequate patient compliance. No foreign body or other cause for vomiting was identified. At the time of ultrasound, the patient was induced with intravenous diazepam (0.5 mg; Pamlin, Ceva Animal Health Pty Ltd., Glenorie, NSW, Aust) and ketamine (10 mg; Ilium Ketamil, Troy Laboratories Pty Ltd., Glendenning NSW, Aust), intubated and maintained using inhalant anaesthesia on isoflurane and oxygen.

The patient developed CPA during recovery from general anaesthesia. CPR was performed for a duration of 15 min and included cardiac compressions, intermittent positive pressure ventilation and intravenous adrenaline (0.1 mg; unknown brand and manufacturer). Following successful resuscitation, abnormal neurologic findings included altered mentation with intermittent vocalization, opisthotonic posturing with extensor rigidity in all limbs, bilaterally reduced pupillary light reflexes (PLR) and palpebral reflexes, and absent vestibulo‐ocular and menace reflexes. Biochemistry and electrolyte changes post‐CPA showed minor deviations from normal reference ranges. The patient was hospitalised for 3 days and treated with intravenous fluid therapy, mannitol (2 g IV; Osmitrol 20%, Baxter Healthcare Pty Ltd., Old Toongabbie, NSW, Aust) and intermittent boluses of diazepam. Amoxicillin and lactulose were also administered for potential portosystemic shunt. The patient was then referred to our emergency and critical care service, part of a university referral hospital, for further management.

On presentation to the emergency service, the patient had a heart rate of 100 beats per minute, pink and dry mucous membranes and a capillary refill time of 1.5 s. Pulses were short and narrow, and the patient was hypotensive with a Doppler blood pressure of 70 mmHg. The respiratory rate was 28 breaths per minute with a normal synchronous respiratory pattern, though transient episodes of apnoea lasting up to 10 s were reported through hospitalisation. Thoracic auscultation was normal. Examination by a board‐certified veterinary neurologist found that the dog had a stuporous mentation, decerebrate rigidity, normal PLR, reduced palpebral and vestibulo‐ocular reflexes and subtle strabismus, particularly when opisthotonic, and absent nasal sensory response. The modified Glasgow Coma Scale (MGCS) was 10/18.^14^ Hypertonic saline (3 ml/kg IV; Sykes Hypertonic saline 7.2% solution, Sypharma Pty Ltd., Dandenong, VIC, Aust) was administered to treat the hypotension, presumed intracranial hypertension and cerebral microcirculatory alterations and maintenance intravenous fluid therapy was instituted (2 ml/kg/h IV Plasma‐Lyte 148, Baxter Healthcare Pty Ltd., Old Toongabbie, NSW, Aust). Norepinephrine (0.05–0.15 μg/kg/min IV; Levophed™ 1:1000, Pfizer Pty Ltd., Sydney, NSW, Aust) was commenced to maintain Doppler blood pressures above 90 mmHg. Dexamethasone (0.1 mg/kg IV q24h; Dexadreson®, MSD Animal Health Pty Ltd., Macquarie Park, NSW, Aust) was administered for suspected cerebral oedema and inflammation, whilst pantoprazole (1 mg/kg IV q12h; Somac®, Takeda Pharmaceuticals Australia Pty Ltd., Sydney, NSW, Aust) was used as a gastric protectant. Due to persistent agitation and vocalisation, multiple doses of diazepam (Ilium Diazepam, Troy Laboratories Pty Ltd., Glendenning, NSW, Aust) were administered before midazolam (0.4 mg/kg/h IV; Hypnovel®, Pharmaco Ltd, Gordon, NSW, Aust) and butorphanol (0.2 mg/kg/h IV; Butomidor®, Ausrichter Pty Ltd, Camperdown, NSW, Aust) were started as constant rate infusions.

**Figure 1:**
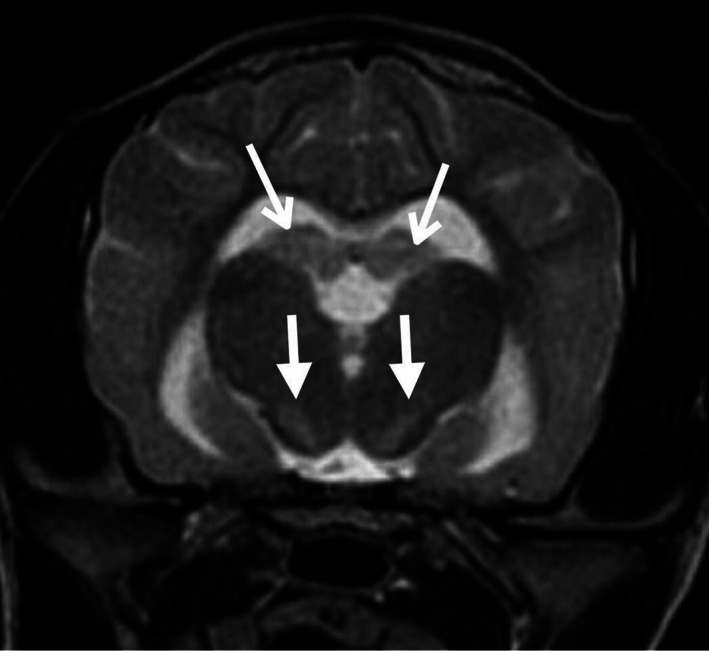
Transverse plane T2‐weighted magnetic resonance image of a dog with global brain ischaemia post‐cardiopulmonary arrest. Note the bilaterally symmetric hyperintensities in the diencephalon in the region of the substantia nigra (closed arrows) and subtle bilateral hyperintensity of the hippocampal tail (open arrows).

**Figure 2:**
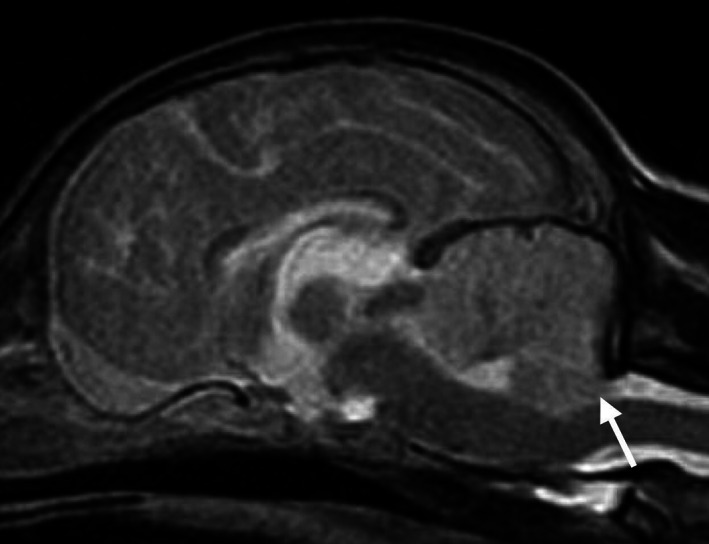
Sagittal plane T2‐weighted magnetic resonance image of a dog with global brain ischaemia post‐cardiopulmonary arrest. There is generalised hyperintensity of the cerebellum which is swollen. The caudal cerebellar vermis is caudally displaced toward the foramen magnum (arrow).

**Figure 3:**
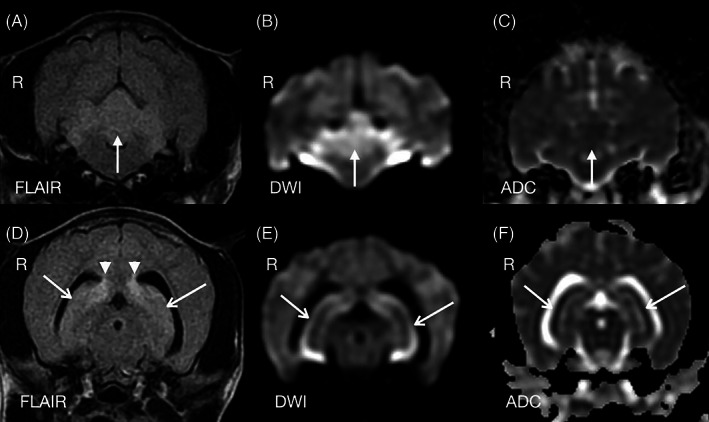
Transverse plane fluid‐attenuated inversion recovery (FLAIR) (a,d), diffusion‐weighted imaging (DWI) (b,e) and apparent diffusion coefficient (ADC) (c,f) MR images of a dog with global brain ischaemia 4 days post‐cardiopulmonary arrest. There are mildly FLAIR hyperintense lesions which demonstrate restricted water diffusion, with DWI hyperintensity and ADC hypointensity. Lesions are depicted in the cerebellum (closed arrows) (a–c) and hippocampus (open arrows) (d–f). Also note peri‐ventricular FLAIR hyperintensity axial to the lateral ventricles (arrowheads).

Four days post‐CPA the patient underwent general anaesthesia for diagnostic imaging performed by a board‐certified diagnostic imaging specialist. Abnormalities on abdominal ultrasound included a mildly enlarged hypoechoic liver, small volume anechoic peritoneal effusion and mild jejunal lymphadenomegaly. A brain MRI was acquired using a 1.5 Tesla magnetic field strength MRI machine (Signa, GE Healthcare, Little Chalfont, Buckinghamshire, England) with a knee radiofrequency coil. Sequences included 3D Fast SPGR T1W, FSE T2W, FLAIR, GRE T2*, and DWI with calculated ADC map. The major MRI findings were symmetric T2W hyperintensity within the basal nuclei, specifically the substantia nigra, the hippocampus (Figure 1), and diffuse hyperintensity of the cerebellum with caudoventral extension of the cerebellar vermis into the foramen magnum (Figure 2), and signs of obstructive hydrocephalus. Both hippocampi and the cerebellum were hyperintense on DWI and hypointense on ADC map, indicating restricted diffusion of water molecules (Figure 3). Low spatial resolution of DWI and ADC images precluded identification of signal changes in other areas of the brain which had T2W hyperintensity. In conjunction with patient history, MRI findings were interpreted as GBI with cytotoxic oedema, cerebellar herniation and obstructive hydrocephalus. Cerebrospinal fluid analysis was not performed due to the presence of cerebellar herniation and associated high risk of morbidity.

Subsequent to the imaging procedures, a percutaneous endoscopic gastrostomy tube (16Fr. PEG Kit, MILA International Inc., Florence, KY, USA) was placed for enteral nutrition. A gastrostomy rather than an oesophageal feeding tube was considered safer due to patient recumbency, probability of aspiration and risk of increased intracranial pressure with a neck bandage. Given the young age of the dog, a *Neospora caninum* indirect fluorescent antibody serology test (Animal Health Laboratory, Tas, Aust) was submitted and later returned negative. Haematology and repeat biochemistry panels showed a mild anaemia (haematocrit 0.30 L/L; RI 0.37–0.55 L/L), reduced creatinine (20 μmol/L; RI 40–140 μmol/L) and elevated alanine aminotransferase [ALT] (341 μ/L; RI 3–83 μ/L). A bile acid tolerance test was normal (fasting 1.7 μmol/L, RI 0–15 μmol/L; post‐prandial 1.5 μmol/L, RI 0–30 μmol/L). The patient was started on phenobarbitone (2 mg/kg IV q12h; Aspen Pharmacare Australia Pty Ltd., St Leonards, NSW, Aust) for seizure prophylaxis and empirical clindamycin (12 mg/kg IV q12h; Dalacin®C, Pfizer Pty Ltd., Sydney, NSW, Aust) treatment for *Neospora* and *Toxoplasma* whilst awaiting results.

Over the subsequent days, the neurologic abnormalities persisted, with repeat MGCS fluctuating between 6–10/18 with changes attributed to variations in vestibulo‐ocular and pupillary light reflex responses. Repeated haematology, biochemistry and electrolyte panels were unremarkable during this time. An arterial catheter for direct blood pressure monitoring was placed on day 5 post‐CPA to facilitate hemodynamic monitoring. Norepinephrine was discontinued the same day, as the systolic blood pressure remained consistently above 100 mmHg without pressor support. Pulse oximetry and repeat venous and arterial blood gases suggested adequate oxygenation and ventilation within the normal range and neither oxygen supplementation nor ventilatory support were deemed required for respiratory optimization.^15^

Seven days post‐CPA, a neutropenia was revealed (1.2 × 10^9^ cells/L; RI 3.0–11.5 × 10^9^ cells/L) prompting the commencement of cefotaxime (30 mg/kg IV q6h; DBL™, Pfizer Pty Ltd., Sydney, NSW, Aust) out of concern for a severe, yet unidentified bacterial infection. The following day a fluid‐filled swelling was noted overlying the left epaxial musculature suspected to be due to leakage from the gastrostomy tube or subsequent to a subcutaneous injection. Cytological examination of a fine‐needle aspirate of the swelling showed degenerate neutrophils with intracellular cocci and a sample was prepared for culture and sensitivity. Early signs of sepsis, the absence of neurological improvement in the face of significant structural cerebral changes ascribed to GBI, and the requirement for surgical debridement of the abscess lead the owner to elect euthanasia that day.

**Figure 4:**
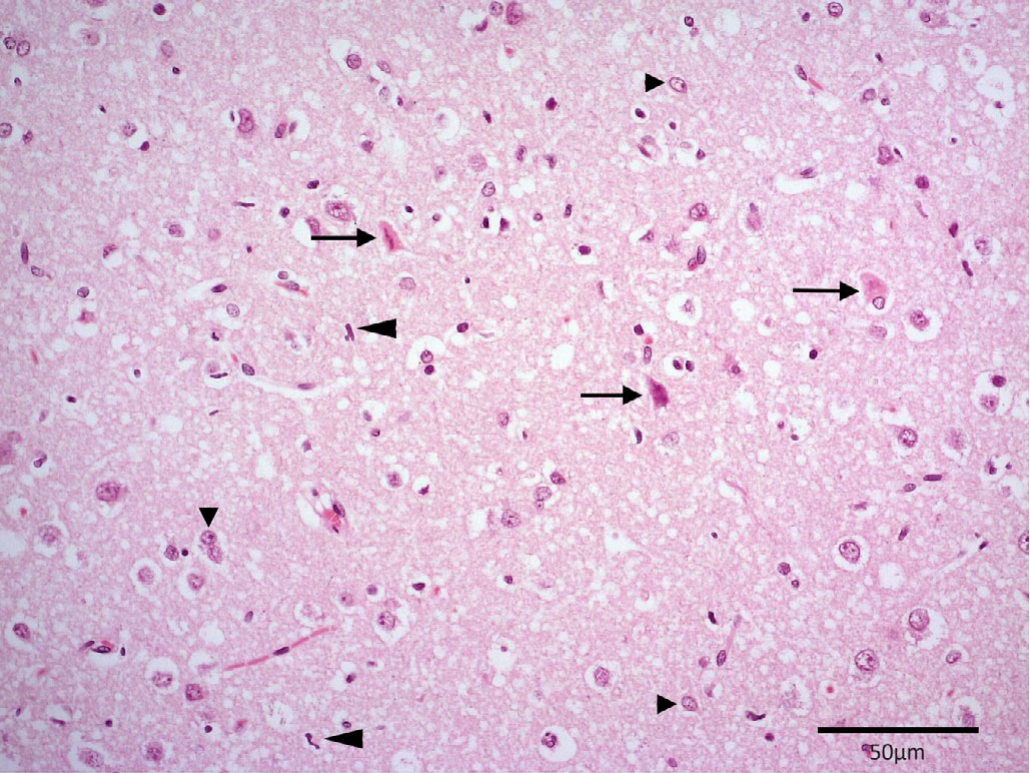
Histopathology of cerebrum in a dog post‐cardiopulmonary arrest. Dead neurons (arrows), scattered migrating neutrophils (long arrowheads), and swollen astrocyte nuclei (short arrowheads). Scale bar 50 μm.

**Figure 5:**
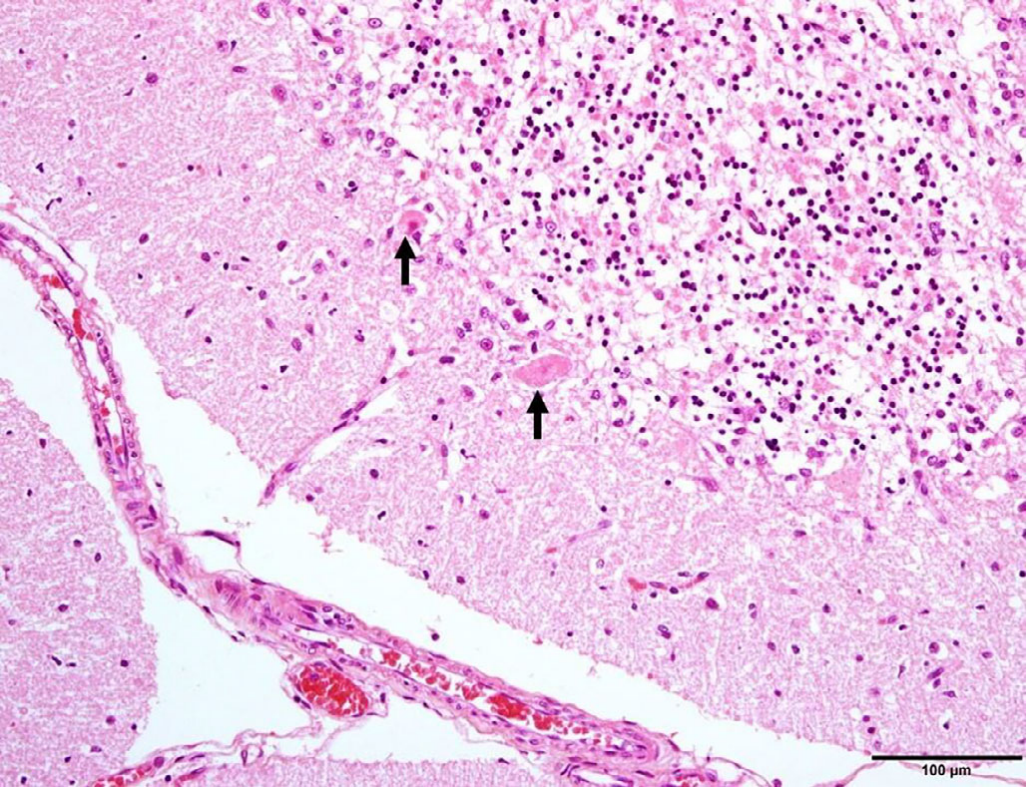
Histopathology of cerebellum in a dog post‐cardiopulmonary arrest. Cerebellum shows pyknosis, loss and vacuolation of the granular layer, with Purkinje cell necrosis (arrows). Scale bar 100 μm.

**Figure 6:**
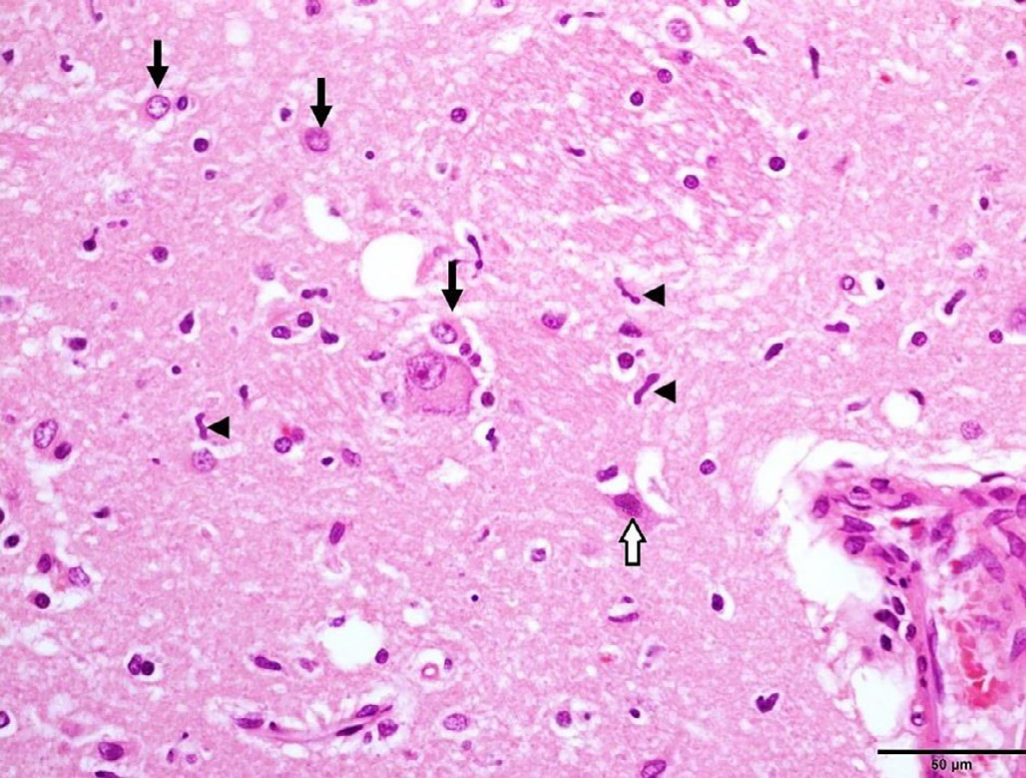
Histopathology of basal nuclei in a dog post‐cardiopulmonary arrest. Neutrophil infiltration (arrowheads), reactive astrocytes (arrows) and dead neurons (open arrow). The included endothelium is reactive suggesting reperfusion. Scale bar 50 μm.

On postmortem examination, the brain was grossly unremarkable. Histopathologic examination identified a moderate number of necrotic neurons and scattered migrating neutrophils within the cerebral cortical grey matter (Figure 4). Necrotic neurons and neuropil vacuolation were present in the hippocampus and thalamus, as well as axonal spheroids in the hippocampus. Sections of the cerebellar cortex showed frank necrosis with extensive loss of Purkinje cells, karyhorrhexis and karyolysis of granular cells and marked neutrophil infiltration (Figure 5). Changes to the basal nuclei included neutrophil infiltration, reactive astrocytes and dead neurons (Figure 6). The overall diagnosis was severe, multifocal, acute to subacute neuronal necrosis. There was also evidence of multifocal, subacute myocardial necrosis on histopathologic examination of the heart. There were no significant changes to other organs identified on post‐mortem. Changes to the brain and heart were consistent with hypoxic injury.

## Discussion

This case report describes the clinical examination, MRI and histopathologic findings in a dog with GBI following naturally occurring CPA. Whilst it is suspected that the GBI experienced in this dog was secondary to CPA, unnoticed complications under general anaesthesia resulting in hypoxic‐ischaemic injury to the brain prior to CPA cannot be excluded.^7^, ^8^ Nevertheless, clinical reports describing post‐CPA brain injury are scarce in veterinary literature, with none written in English linking ante‐ and postmortem findings.

In comatose humans with out‐of‐hospital cardiac arrest, two thirds of patients will die from hypoxic‐ischaemic brain injury, mostly from withdrawal of life‐sustaining treatment after prognosticating a poor neurologic outcome.^6^, ^16^ Scoring systems such as the Cerebral Performance Category are used to report neurologic outcome after CPA in humans and typically stratify according to whether independence, work and social capacity and an overall return to an acceptable life at home is achieved.^16^ Predictors of neurological outcome are most often evaluated for their accuracy to predict a poor outcome, as the critical clinical question is foremost whether to withdraw life‐support or not.^6^, ^16^ As a single prognostic factor is often not sufficient to predict a poor outcome with high certainty, resuscitation guidelines in people suggest using a multimodal approach to neuroprognostication in the post‐CPA period – a combination of clinical examination, neuroimaging, electrophysiologic testing and biomarkers.^6^, ^16^ Unfortunately, electrophysiologic testing (i.e., electroencephalogram [EEG] patterns, short‐latency somatosensory evoked potentials [SSEP]) and cerebral biomarkers (i.e., neuron specific enolase and S‐100B) are not yet readily available in veterinary medicine. Absence of brainstem auditory evoked response (BAER) or brainstem reflexes such as the caloric reflex could indicate severe brainstem dysfunction and brain death.^17^, ^18^ But current evidence suggests that it is unclear whether the use of auditory evoked potentials is suitable to predict poor neurological outcome after CPA in humans,^19^ and the use of caloric reflexes have rarely been reported in the post‐CPA population.^20^ Future studies on post‐CPA BAER and caloric reflex in dogs and cats would fill an important knowledge gap. Currently, this leaves physical examination and neuroimaging as the most clinically relevant means to neuroprognosticate in small animals.

Extrapolating from recommendations in people, it is reasonable to recognize that poor neurological outcome is difficult to conclusively predict during the first 24–72 h after resuscitation.^2^ Physical examination should be interpreted considering possible confounders, such as sedation.^2^ In people that remain unconscious, absence of pupillary and corneal reflexes in this time period are highly predictive of poor neurologic outcome, especially if combined with continuous and generalized myoclonus (i.e., involuntary jerks) for at least 30 min.^2^, ^6^ At presentation to our hospital 3 days post‐CPA, the predominant neurologic signs in this dog included a stuporous mentation, decerebrate rigidity and intermittent apnoea, indicating a rostral brainstem injury, but PLR and corneal reflexes were intact and no myoclonic jerks were observed. Areas of the brain commonly susceptible to hypoxic‐ischaemic injury include the cerebral cortex, cerebellum, hippocampus and thalamus, with the brainstem reportedly more tolerant to such injury in humans.^21^ Neurolocalisation to the brainstem may indicate more severe ischaemic injury, though other veterinary case reports of GBI have neurolocalised lesions to the brainstem and these patients survived.^7^, ^8^, ^9^

Current literature describing post‐GBI neurological deficits in dogs is scarce, limited to individual case reports or small case series. Common abnormalities in all these patients included ataxia, seizures, blindness, pupillary size changes and altered mentation from obtunded to stuporous. Bellis et al.^11^ and Palmer and Walke^12^ describe three dogs with neurological abnormalities post‐CPA. Clinical signs were variable across all three and ranged from opisthotonus, paddling, convulsions, vocalisation and pupillary changes. Outcome was variable; one developed central diabetes insipidus but survived to discharge after 8 days, one was euthanased and one died. Although some of the signs reported are similar to those observed in this case, observations are too scarce to draw prognostic conclusions. Nevertheless, as in humans, these reports indicate that the presence of initially severe neurological abnormalities even if persisting for several days does not preclude an acceptable functional outcome.^6^

In people, patients with a Glasgow Coma Scale value of ≤8 (i.e., comatose) or a Glasgow Motor Score of ≤3 (i.e., abnormal flexion to painful stimuli or worse) at 72 h post‐CPA require systematic approach to prognostication, as a good functional outcome is uncertain.^2^, ^6^ Our patient had a persistently low MGCS with no clinically tangible improvements in neurologic ability throughout hospitalization. In this case, the MGCS was used to monitor trends in neurologic function. However, it was designed to evaluate the severity of brain injury following trauma in dogs and has not been validated for prognostication in dogs post‐CPA. The MGCS evaluates three neurologic categories: motor activity, brainstem reflexes and level of consciousness. In dogs with trauma it was found to provide prognostic information, with a 50% probability of non‐survival with a score of 8/18,^14^ and 85% sensitivity and 73% specificity for predicting death with a score ≤11/18.^22^ In our case, a MGCS between 6–10 suggested severe neurologic dysfunction and the outcome was in line with what was expected for dogs with traumatic brain injury. Both Lee et al.^9^ and Bellis et al.^9^ utilized the MGCS; their patients' scores improved over time and both dogs survived. One clinical study adapted a 0–400 neurologic scoring system from a post‐CPA swine model, with all nine dogs alive after 1 h showing improved scores as time progressed.^23^ Neurologic deficit scores may allow objective serial monitoring of functional neurologic recovery but need to be validated for post‐CPA cases before using them for clinical decision making.

In contrast to physical examination, brain imaging with CT or MRI is not dependent upon sedation and allows more objective assessment.^6^ In people the presence of diffuse and extensive anoxic injury on either imaging modality, alongside respective physical examination findings, supports a poor neurological outcome.^2^ Although the prognosis based on physical exam at 72 h post‐CPA was not favourable in this dog, the owners elected to gather as much objective information as possible to aid with decision making; brain imaging was the preferred diagnostic modality to obtain such information. In the case presented here, the MRI identified GBI injury to the substantia nigra of the basal nuclei, the hippocampus and cerebellum. DWI and ADC images highlighted the lesions within the hippocampus and cerebellum. MRI permits noninvasive structural evaluation of the brain following a suspected ischaemic event. When standard brain MRI protocols based on T1W, T2W and FLAIR sequences are used, MRI has low sensitivity to correctly distinguish ischaemic brain disease from neoplastic and inflammatory brain disease in dogs.^24^ MRI sequences that measure the diffusivity of water, such as DWI with a calculated ADC map, are the most sensitive and specific to evaluate cerebrovascular accidents in dogs.^24^ Ischaemic brain injury results in cytotoxic oedema, characterised by increased diffusion of water from the extracellular to intracellular space and reduced diffusion within the cell. Restricted diffusion of water molecules appears as hyperintensity on DWI and hypointensity on the ADC map.^24^

The cerebellum and substantia nigra have not been described as affected on MRI in previous canine case reports of GBI,^7^, ^8^, ^13^ and only one case mentions bilateral hippocampal lesions.^10^ In a dog that developed MRI findings of GBI after bite wound surgery, Lee, Park and Kang^9^ reported T2W hyperintensities in the olfactory bulb, and the frontal, parietal and temporal lobe, but not specifically the hippocampus. Timm et al.^8^ noted basal nuclei lesions but in the rostral caudate nuclei not substantia nigra. Hippocampal, cerebellar and basal nuclei lesions have been reported in human MRIs with GBI, the former two associated with poor clinical outcomes.^25^, ^26^ The patient in the present case did not demonstrate the often‐reported cerebral cortex MRI lesions, however this cortex‐sparing distribution has also been reported in GBI‐affected humans.^25^, ^26^

Studies also indicate that changes on DWI and ADC map can provide prognostic neurologic information in post‐CPA humans.^25^, ^26^, ^27^ Current human guidelines recommend DWI sequences to be obtained 2–5 days after cardiac arrest to help predict neurologic outcome.^16^ Different thresholds for predicting poor neurologic outcome post‐CPA such as whole‐brain ADC, the proportion of brain volume with low ADC or the lowest ADC value in specific areas of the brain have all shown promise.^16^, ^27^ DWI and ADC maps in experimentally‐induced CPA models using dogs^28^ and cats^29^ found ADC could be used to monitor progression or reversal of ischaemic brain injury. In humans, the absence of DWI abnormalities within 1 week of CPA is suggestive of good neurological outcome.^16^ Future work should encourage the routine use of these sequences post‐CPA to better understand the pattern, severity and progression of brain injury ante‐mortem, building our knowledge base to better neuroprognosticate for these patients. CT can be considered to identify cerebral oedema post‐CPA as it is more readily available, financially viable and faster than MRI, and has been used to neuroprognosticate post‐CPA in humans.^16^ However, its sensitivity to detect ischaemic brain injury is reduced compared to MRI,^21^ especially in dogs and cats in which oedema is more difficult to identify with CT than in people.^24^

Histopathology results in this dog confirmed neuronal necrosis in the areas identified on MRI, as well as the cerebrum and thalamus, and follow a pattern of susceptibility that is known to affect certain areas of the brain. Ischaemia and reperfusion lead to post‐CPA brain injury, with most damage occurring in the reperfusion stage. The pathophysiology involves complex, overlapping processes and has been well described elsewhere.^7^, ^30^ Specific areas of the brain, namely the cerebral cortical layers 3–5, the hippocampus CA1 region, the cerebellar Purkinje cells and the basal nuclei are particularly vulnerable to ischaemia‐reperfusion injury.^21^, ^30^ Two hypotheses – the excitotoxic neurotransmitter and the free radical hypothesis – attempt to explain why. They state that selectively vulnerable neurons either contain large amounts of glutamate and excitatory receptors or are deficient in the antioxidant glutathione peroxidase and are surrounded by iron‐rich oligodendrocytes. Following ischaemic injury, these neurons are prone to excitation‐related calcium release or free radical formation, respectively, making them prone to cellular damage.^30^ It is likely that both hypotheses are not mutually exclusive and together contribute to the death of these neurons. In this case, all regions known to be vulnerable to ischaemia showed some level of neuronal necrosis. Importantly, most of the affected areas were apparent on MRI 4 days prior to post‐mortem.

## Conclusion

This case report presents the clinical, MRI and histopathology findings in a dog with GBI secondary to general anaesthesia and CPA. A lack of significant neurological improvement and persistently low MGCS characterized the 8 days post‐CPA in this patient. Standard MRI sequences identified anoxic injury to multiple regions of the brain, including the hippocampus, cerebellum and substantia nigra of the basal nuclei. DWI and ADC maps confirmed cytotoxic oedema in the hippocampus and cerebellum, areas associated with a poor neurological outcome in humans. There was good correlation between MRI and histopathological findings in the present case, suggesting that MRI may be used as a tool to determine the extent of GBI following CPA in the dog. Further studies are required to investigate the validity of physical exam‐based neurologic scoring systems, the use of quantitative MRI measures such as ADC, and the timing of these entities to provide neuroprognostic information post‐CPA.

## Conflicts of interest and sources of funding

The authors declare no conflicts of interest or sources of funding for the work presented here.
